# Self images: an empirical enquiry into Rembrandt’s self-portraits

**DOI:** 10.3389/fpsyt.2026.1631373

**Published:** 2026-04-14

**Authors:** Eric C. Bettelheim, Jingyi Liu, Paola Dazzan, Federico Turkheimer

**Affiliations:** Department of Neuroimaging, Institute of Psychiatry, Psychology and Neuroscience, King’s College London, London, United Kingdom

**Keywords:** depression, neuroscience of art, painting, quantitative methods, Rembrandt, self portraits, stylistics

## Abstract

Many have speculated that events of personal and financial loss in the life of Rembrandt van Rijn (Rembrandt) caused depression and that this is revealed by examination of his work particularly self-portraits painted in old age. Some report detecting various physiological diseases associated with aging, including vision impairment, which may have affected his mood and work. Aging and neurodegenerative disease which often accompanies it, are both associated with depression. Depression is characterised by visual deficits including perception of reduced contrast and colour. Age-related neurological disorders are associated in artists with reduced complexity. Recent advances in imaging and computer technology make it possible to empirically examine changes in artistic style which can contribute to understanding artists’ physical and mental health. Previous studies have identified associations between adverse events in artists’ lives and altered contrast and colour in their self-portraits. In the current study changes in contrast, colour and fractal dimension were measured in the entire corpus of Rembrandt’s painted self-portraits and portraits to determine whether changes in style indicate depression, cognitive decline, or neurological disease and whether differences in style can be detected between self-portraits and portraits of related and unrelated others. Productivity was also examined as an indirect indicator. The results suggest that it is unlikely that Rembrandt suffered from unipolar or bipolar depression, age-related cognitive decline, or neurodegenerative disease. The data are consistent with someone experiencing episodes of low mood associated with normal grieving and adversity followed by resilient recovery. There is evidence of a gradient in saliency and complexity between self-portraits and related and unrelated portraits and of a ‘late’ style identified by leading art historians consistent with macular degeneration.

## Introduction

Rembrandt van Rijn (1606-1669), ‘Rembrandt’, was born in Leiden into a tolerant, ‘middle class’ family and enjoyed a comfortable and stable childhood. He received a thorough classical and religious education. His decision to become an artist rather than attend university was supported by his father who enrolled him as an apprentice with two well-known artists. Rembrandt was ‘discovered’ in 1629 by the secretary to Prince Frederick Hendrik Holland’s leader and a patron of the arts. Rembrandt rapidly became recognized as among the greatest painters of his day soon moving to Amsterdam and becoming commercially successful in a competitive marketplace. His mastery in conveying human emotion was recognised from the beginning of his career (1–3). He painted more self-portraits than any artist before him, depicting himself in early adulthood, middle and old age.^1^ More than one scholar has seen them as constituting an autobiography (4, 5). He also painted numerous portraits on commission and of family members and friends. Self-portraits and portraits represent nearly half of his painted oeuvre and were painted throughout his career providing the opportunity to examine changes in style longitudinally (6).

The self-portraits Rembrandt produced in his last decade are seen by many as the finest images of old age ever painted (7–9). It is primarily based on these, a few etchings, and a series of adverse life events, that some have concluded that Rembrandt suffered unipolar or bipolar depression (10, 11). The events include: the death of his father in 1630, of three children shortly after birth (1635, 1638 and 1640), of his mother (1640), of his wife (1642), his bankruptcy in middle age (1649-1658) and resulting reduced financial and social circumstances, the death of his mistress (1663) and the death of his only surviving son (1668). (3, 12, 13). Such life events, as well as old age itself, are risk factors and potential triggers, individually and cumulatively, of depression (14–16)^2^.

It is important to see such events in their historical context. In 17^th^ century Holland infant mortality was approximately 40% (17) and the plague, which killed his wife, his mistress and likely his son, was a common source of adult mortality (18, 19). In this early stage of capitalism, bankruptcy was already well catered for by specialised institutions. Bankruptcy among artists was not uncommon and the terms of Rembrandt’s were not unduly harsh allowing him to continue his work albeit under straitened circumstances. Following his bankruptcy and as he aged, Rembrandt’s social circle declined but he continued to be supported by his son and mistress, maintain friendships, and engage with patrons. He continued to work until his death (20–22).

Just as it is important to put the events of Rembrandt’s life in their historical context one must use modern criteria of diagnosis of physical and mental illness with caution as one is inevitably handicapped by the very different conditions in the past. Rembrandt lived in the pre-modern period in which concepts of mental and the prevalence of physical illness were very different from our own (23–25). It is important when attempting to describe the mental state of figures in the past to be conscious of the context in which they lived when applying modern insights (26). Posthumous diagnosis is problematic even when there is considerable evidence of recent vintage (27, 28). It is all the more difficult when dealing with someone who died centuries ago.

Rembrandt’s life and work has been the subject of extensive study, beginning in his lifetime and extending to the present day (29). There is, however, relatively little personal documentation. The few letters in his hand to survive were written to patrons on business matters. The remaining documents by or related to him are mainly fragmentary or related to financial and legal matters (22). There are contemporary descriptions of him, his studio and his working habits but we have little idea of what he thought or felt except in regard to the high value he put on his work. There are no contemporary accounts of Rembrandt suffering any physical or mental illness or cognitive decline. There are no indications of unusual physical or of mental illness in Rembrandt’s family. Despite the paucity of evidence, the number and quality of his self-portraits have encouraged scholars to make diagnoses both physiological and psychological.

## Somatic and mental illness

Rembrandt lived beyond the average span of his contemporaries (30) dying naturally of old age. A number of scholars have sought to identify somatic illnesses from examination of the self-portraits, particularly diseases associated with aging, vision and depression (31, 32). Physical illness is associated with depression (33, 34) and depression is common in old age. Approximately one third of European older people (65+) experience depression (35) and a similar proportion is found globally (36). Deterioration in vision, particularly macular degeneration, is associated both with aging and depression (37). There is no evidence that Rembrandt wore spectacles, although they were widely available.

The extraordinary detail in Rembrandt’s depictions of himself in old age has encouraged meticulous examination of his facial features^3^ resulting in conclusions that he suffered from a variety of diseases including: temporal arteritis, hyperthyroidism, rosacea and rhinophyma, cataract, macular degradation, glaucoma, unilateral strabismus, presbyopia, xanthalasma, pinguecula, arcus senil and yellow-brown vision (32, 38, 39). These analyses have been reviewed in detail by disease specialists and found unreliable (40–43). The reliable evidence from such examination of the self-portraits is that Rembrandt suffered physiologically from no more than normal aging which may have included macular degeneration and presbyopia (38, 44, 45). Given that he lived into old age Rembrandt was at risk of cognitive decline and neurodegenerative disease however no symptoms were noticed by contemporaries. One of them described him 5 years before his death as “hale and hearty” and “in full use of his mind, memory and speech” (2).

### Depression

The persistent suggestions are that Rembrandt suffered from depression following the death of his wife, during and after his bankruptcy and in old age compounded by somatic disease, including vision impairment, and that this is reflected in his self-portraits (11, 32, 46). Many perceive sadness in Rembrandt’s self-portraits given the “unsparing” honesty with which he portrayed aging (3). Scholarly, medical, psychiatric, and journalistic analyses of these and other images have concluded that at various points in his life Rembrandt suffered from depression. The evidence for this conclusion is principally subjective and reliant on selective use of images and events. For example, a review of one etching of St Jerome and one of an unidentified somber sitter are said to demonstrate depression (10). It is suggested that these images and a decline in the number of etchings produced after his wife’s death indicate a period of depressed mood. Although the etchings on which the diagnoses of unipolar depression were based (11) are of melancholy subjects they are not images of Rembrandt. Whenever Rembrandt included his self-image in any work, he ensured that he was notably recognisable and never did so in the pose of melancholy. If Rembrandt noticeably suffered from melancholy he and his contemporaries would have been able to recognize and name it and he could certainly have depicted himself as such. Neither he nor any of his contemporaries ever so described him. The many other images which he produced during this period, etched, painted and drawn, are not considered.

The same observer then extended the proposition to bipolar depression based on Rembrandt’s subsequent period of high, ‘manic’ spending (11). Rembrandt spent freely following his marriage acquiring both an expensive house and a collection of art and rare objects for which he paid high prices. His mismanagement of financial matters during the 15 years following his wife’s death ultimately resulted in his bankruptcy (2, 20). Yet his extravagance extended for years and was not characterised by the relatively brief periods, measured in weeks, of manic episodes (47) and his spending related overwhelmingly to his artistic enterprise.

There have also been efforts, including by plastic surgeons, to analyse the details of his face, particularly its musculature, skin condition and his eyes although none of the characteristics noted are biomarkers or reliable indicators of depressive disorder (32, 48). The only previous attempt to objectively measure stylistic variation in the self-portraits and correlate it with Rembrandt’s life events measured differences in brightness and darkness believed to indicate depressed mood. The results were inconclusive and flawed by reliance on photo-electric analysis of reproductions (49).

### Depression and vision

In Rembrandt’s day depression would have been described as melancholia. Indeed, it was fashionable for creative people to be seen as melancholic. Burton’s (50) work on the subject was well known as was Durer’s etching ‘Melencolia’ which established the now emblematic pose which Rembrandt used in several works (51). If Rembrandt noticeably suffered from melancholy he and his contemporaries would have been able to recognize and name it and he could certainly have depicted himself as such. Neither he nor they ever did so. Melancholia is now encompassed by modern definitions of depression (52).

Both anecdotally and experimentally, individuals with depression see the world as darker with ambient light diminishing as the disease progresses (53–55). There appears to be a bi-directional relationship between feelings and perception. Those feeling sad perceive things as dim in comparison to those feeling happy who see things as brighter (56). There is compelling evidence of visual abnormalities in depression (57). Medicated and unmedicated, first episode and chronic sufferers of major depression (MDD) have reduced visual acuity, lower retinal contrast gain, lower contrast sensitivity, and altered surround suppression which correlate with disease severity (53, 54, 58–63). Those suffering from bipolar disorder (BPD) reveal similar deficits (64, 65).

Investigations of the retina using optical coherence tomography (OTC) have found reductions in thickness of the retinal nerve fibre layer (RNFL), ganglion cell layer (GCL) and inner plexiform layer (IPL) and thicker choroid levels in MDD and BPD patients as compared to healthy controls (66–68). Dysfunction in these layers is evidenced by decreases in a and b wave amplitudes measured by electroretinograms (ERG) (69). Pattern electroretinogram (PERG) studies, which measure the activity of retinal ganglion cells, whose axons make up the optic nerve, find increases in P50 and N95 implicit time. (70). ERG and PERG studies find rod and cone dysfunction in both medicated and medication naïve patients (71). In mild cases there appears to be no significant differences in the retina compared to healthy controls but in more severe MDD and in BPD the differences are significant (67, 72–74). A global study of retinal function in acute MDD found delayed, hypoactive central retinal signalling in photoreceptors and bipolar cells together with hyperactivity in the peripheral retina consistent with subject reports of dimness in their surroundings (75).

Individuals with depression seeing the world as darker correlates with reduced contrast gain control evidenced by lower retinal PERG and EEG amplitudes and higher contrast-detection thresholds (53, 54, 58, 59, 62, 63, 76). This appears related to altered mediation by dopamine and serotonin (53, 55, 60). Those suffering from MDD have significantly reduced levels of dopamine which is critical to contrast gain control (77). Dopamine hypofunction in the retina alters the behaviour of amacrine, horizontal, interplexiform and ganglia cells (78). Rods are dopamine dependent and changes in dopamine levels affect cognitive vision processing (79). Those with MDD show reduced amplitude in visual evoked potential (VEP) indicating that the dysfunction extends beyond the retina to the occipital cortex which is innervated by the mesocortical dopamine system (59). Deficits in surround suppression also occur in the primary visual cortex (61).

There is consistent evidence that colour perception is impaired in both MDD and BPD (80, 81). Compared to healthy controls depressed people are far more likely to select grey as representative of their mood, in contrast to yellow preferred by controls and associated with positive feeling. MDD patients also prefer desaturated colours and there is evidence of reduced colour in patients’ art (82–84). In melancholic depression there is both dimming of the environment and a reduced sense of colour related to retinal dopamine dysregulation (85). Hypo-dopaminergic states in the retina are associated with poorer discrimination of red as well as blue colour saturation (86). There is evidence from cocaine-dependent, tricyclic treated depression, Tourette’s, Parkinson’s and Huntington’s patients that low dopamine levels cause deficits in short-wave, blue-yellow cone function (87–92). This is also the case in normal aging (93). This may be a result of altered communication between blue photoreceptors and horizontal cells indicated by abnormal blue cone ERG measurements (91, 94).

### Aging and vision

The prevalence of depression in old age approaches 30% (95). Old age is often a period of social isolation and loneliness which are related to physiological and psychological deterioration. Loneliness is correlated with depression but while older people can experience social isolation, sparse or inadequate relationships, loneliness and feelings of loss, they are not directly related. Isolation alone does not necessarily imply loneliness or the likelihood of mood disorder (96, 97). While many cognitive functions such as verbal and numerical abilities and general knowledge continue unimpaired into healthy old age others, including processing speed, reasoning, memory, and executive functions, often experience a decline. Healthy individuals with higher education and occupational achievement develop a ‘cognitive reserve’ which assists in preserving cognitive capacity (98).

There is evidence that, in contrast to general declines in cognitive function, in normal aging reduced suppression and increased functional connectivity of the default mode network (DMN) enhances creativity in older people in comparison with younger adults. It is also associated with increased resilience (99–101). Altered neurological architecture, cognitive reserve and increased connectivity may be reflected in the work of healthy aged artists who demonstrate continued vitality, increased mastery and an ability to convey life’s deeper meaning. This helps explain the frequency of masterpieces being painted in old age (7, 102). Exceptional artists often demonstrate enhanced creativity in old age (103). This contrasts with other fields of creativity such as mathematics and science in which creativity is highest in early to mid-adulthood (104).

Distinct patterns of fractal dimension have been found in healthy aging painters (Monet, Chagall, Picasso) as compared with those who suffered from degenerative disease (Dali, de Kooning, Morrisseau, Brooks). The former demonstrate increasing complexity with advancing age and the latter declining levels with the advance of disease (105). Several neurological conditions, in addition to depression and normal aging, are characterised by reduced levels of dopamine and low levels of blue cone response (72, 91, 94, 106). In Alzheimer’s disease and attention deficit hypoactivity disorder hypodopaminergic conditions are associated with reductions in tritan (blue-yellow) spectrum perception (86, 107, 108). This is consistent with shortwave cone dysfunction. Shortwave cones are responsible for processing blue colour and highly sensitive to dopamine dysregulation (109).

Among the manifold physiological effects of aging there is both a longitudinal and a bi-directional association with vision impairment (110). Visual deficits due to neural degeneration are common in the elderly often the result of neural and macular degeneration, diabetic retinopathy, glaucoma and cataract (37, 111–115). The effects include reduced spatial and temporal contrast sensitivity and resolution, slower visual processing, restricted visual field, reduced spatial acuity, presbyopia and reduced peripheral vision. Such changes appear related to thinning of the RNFL, loss of macular volume, reductions in thickness of the GCL, degeneration of the optic nerve, loss of volume in the IPL, inflammation, swelling of photoreceptors and microvascular abnormalities including increased choroid thickness (116, 117).

Aging is also accompanied by significant loss (≈30%) of rod density reducing perception of contrast, contrast sensitivity, and motion particularly at the periphery (118). Deterioration of contrast perception begins with high spatial frequencies in middle age and then extends to all frequencies by age 60 (119). Those who are socially excluded perceive their environment as darker and compensate by seeking brighter conditions (120). Symptoms include impaired vision in scotopic conditions, reduced contrast sensitivity at both low and normal levels of luminance and reduced motion perception. All of these symptoms are consistent with dysfunction in the magnocellular pathway and accelerated rod loss (111, 121–124). Deterioration in older healthy subjects is found in both the parvocellular and magnocellular pathways but predominately in the latter (125). Contour integration and surround suppression are also significantly reduced in the aged (126–128).

Colour perception and discrimination in healthy adults begins to deteriorate from age 50 and accelerates thereafter. Macular degeneration, the most common age-related deficit, causes loss of central visual acuity due to macular thinning and deterioration of the retinal pigment epithelium. The latter includes cone loss which generates dysfunction in processing the tritan and red-green axes altering colour vision. Sensitivity to shortwave (blue-violet) colours is reduced and there is a preference for yellow backgrounds particularly in scotopic conditions. This is the result of receptor and post-receptor dysfunction and yellowing and increasing density of the lens reducing light reaching the retina. The effect is to reduce shortwave, with a consequent increase in the proportion of long wave, light perceived (45).

### Fractals

Mandelbrot (129) discovered that there is a random, chaotic element in the regularity of natural patterns which he called fractals. Fractals can be seen as emergent, non-linear, spatial and temporal patterns of both chaotic natural phenomena and of self-organising systems such as the brain, which can be measured empirically (130, 131). This form of regularity can be seen to follow a log power law of fractal dimension ‘D’ varying from 1 to 2 or 2 to 3 depending on whether 2 or 3 dimensions are being measured. This variable can be seen as a measure of spatial order-disorder (number of regularities) and simplicity- complexity (number of elements). D increases as the overall structure increases in fine spatial detail reflecting the relative complexity of the object (129). The D value is essentially a ratio of filled, repeating structural detail to empty space. Fractals thus provide a statistically reliable method of quantifying visual complexity by measuring the relative amount of coarse and fine structure in any image including art images (132–135).

There is considerable evidence that the eye and brain have evolved to perceive and prefer the statistical properties of natural scenes which are used to interpret and optimise sensory data (136–140). Features of the natural environment have fractal values in the mid-range of 1.3-1.5 and humans are attuned to these levels (141–143). Human eyes follow a search trajectory measured by saccades with a D value of 1.4-1.5 indicating a resonance between the visual system and natural scenes and objects (144, 145). Retinal adaptation to contrast also follows a fractal pattern (146). Nearly identical fractal patterns and patterns of activity are found throughout the visual system. This includes neurons of the retina, lateral geniculate nucleus and occipital cortex whose dendritic trees use fractal patterns in the range of 1.33-1.51. This optimises efficiency in signal transmission (147–149).

EEG responses confirm that natural images with mid-range D values are appealing, reduce stress and stimulate calm attention: high alpha in the frontal lobes and high beta in the parietal area (150–153). Visual preference for mid-range fractals extends across categories (natural scenes, moving objects, mathematical patterns and paintings) and is associated with beauty in nature and in humans. It is already apparent in children and extends across cultural boundaries suggesting it is a trait (133, 143, 154–158).

These scale invariant, sparse, regularities of spatial, luminant and chromatic natural characteristics are also reflected in art (136, 159, 160) and are an important element in observer preferences (161). Mid-range fractal values are aesthetically appealing. Artists unconsciously depict faces with fractal values in the mid-range (1.3-1.5) rather than as found in real faces (162, 163). Departures from this norm, in either direction, are disturbing in painted and other images. Spatial frequencies in art significantly higher than those found in nature are associated with triggering epileptic seizure and migraine in susceptible individuals and aversion in healthy subjects (164). Lower and significantly higher values are less appealing in both synthetic images and in art (150, 158, 165, 166). There appears to be a preference for some natural images and abstract patterns with D values somewhat above 1.5 (133, 167).

Fractal analysis has illustrated changes in complexity which correspond to stylistically defined periods in artists’ careers (168). Jackson Pollack was found to have an identifiable pattern of fractal development in particular periods. Complexity in his work increased over time from a D value close to 1 to 1.72 in his ‘drip’ paintings. This is significantly above the preferred middle range of 1.3-1.5 (151, 169, 170). This may account for early negative critical and public reactions (171). His contemporary Mondrian moved in the opposite direction from early naturalistic paintings with a fractal dimension of 1.7 to abstract paintings consisting of lines of varying thickness with fractal values approaching 1 (165, 172). The paintings of artists suffering from neurological disorder in old age reveal declining D levels as compared with those of healthy artists which increase with age (105).

In MDD deviant fractals are associated with inflexible, rigid and less spontaneously adaptive, rhythms and periods of hand motion and physical activity (173, 174). Non-linear fractal analysis of EEGs in MDD patients found higher levels of complexity compared with healthy controls. This is consistent with neuroimaging studies revealing hyperactivity in frontal and parietal areas (175). BPD, in contrast, is characterized by pre-frontal grey matter reduction and hypoactivity commensurate reduced fractal dimension as compared with healthy controls (176). Normal aging is characterized by declines in subcortical and cortical fractal values (177, 178) and reduced dendritic arborisation (179). Higher fractal values in white matter correlate to greater cognitive fluidity in old age (180).

### Hypotheses

Uniquely in art, in self-portraits the subject and object are the same. Higher salience is attributed to, and there is greater retention in memory of, self-related matters and images compared to those related to others (181–183). It is therefore plausible that self-portraits are more likely to reveal changes in the artist’s state of mind than other subjects. Previous research has identified stylistic changes in artist’s self-portraits associated with changes in psychological and physiological states. Changes in contrast in the self-portraits of Vincent van Gogh correlated with periods of alcohol-related psychosis (184). Changes in colour associated with episodes of physical pain and rage were detected in the self-portraits of Frida Kahlo (185). Increases in contrast, colour and fractal dimension during psychotic episodes have been found in the self-portraits of Edvard Munch (186).

The present study examined changes in contrast, colour and fractal dimension in the self-portraits of Rembrandt. The goal was to determine if these changes correlated with adverse events associated with depression or would be expected due to neurological or visual deterioration associated with aging. His portraits were examined as a control and to determine if self-portraits differed from portraits of others generally and if there were variances between commissioned portraits and those of people related to him (family and friends). Productivity was also examined as an independent variable.

Given the evidence of physical and psychological health and normal aging in Rembrandt’s life it was hypothesised that changes in contrast, colour and fractal dimension would not indicate depression, cognitive decline or neurological disease. It was further hypothesised that changes in the paintings would be consistent with transient low mood in adversity and with age-related macular degeneration. Specifically, it was predicted that: (i) contrast would not alter, (ii) the use of colour, except in the tritan spectrum, would not vary, and (iii) that changes in fractal dimension would not be correlated with adverse events nor indicate neurological or cognitive decline. Finally, it was hypothesised that portraits of related and unrelated people would vary from self-portraits and from each other.

## Methods

### Materials

Rembrandt’s style and technique have been the subject of a decades long multi-disciplinary study devoted to authentication of his works known as the Rembrandt Project. It constitutes the most extensive examination of any artist’s oeuvre. The results are published in ‘A Corpus of Rembrandt’s Paintings’ (29). Although a wide variety of scientific techniques were utilised in the study computer-based analysis of stylistic elements was not among them as these techniques only recently became available. The former director of the Rembrandt Project revised the Corpus and attributed a total of 324 paintings to Rembrandt (6). It is on this catalogue that the present study relied for both authenticity and chronology.

Forty-one of Rembrandt’s paintings are self-portraits constituting some 12% of his painted oeuvre. Eight have suffered physical deterioration. These were included as tests of the three variables did not produce anomalous results. Seven images of Rembrandt in historical and biblical paintings are excluded. 116 portrait paintings were considered consisting of 84 individual portraits of unrelated sitters, 11 group portraits of two or more unrelated individuals, 16 of family members and 5 of friends (‘related others’) (6, 187). One portrait of an unrelated individual was excluded due to poor condition. Character studies, known as ‘tronies’ were excluded as were images of models. Portraits so defined represent 35% of Rembrandt’s painted oeuvre. In total 156 paintings, 48% of Rembrandt’s painted work, were included. All are painted in oil on wood panel or canvas (188). Works on paper, including self-portraits, were not considered except in regard to overall productivity.

All the images examined are high resolution (>300 PPI) digital images downloaded wherever possible directly from the institutions which own the original paintings. Where this was not possible high-resolution images were obtained from open sources. Most institutions provided images in Tag Image File Format (TIF or TIFF) which preserves image quality regardless of copying. Some images were provided in Portable Network Graphics (PNG) format which is equivalent to TIFF in all relevant respects. The remaining images were obtained in Joint Photographic Experts Group (JPEG) format. Images obtained in JPEG and PNG format were converted to TIFF using a publicly available on-line service (xconvert.com) to ensure both consistency and stability of the images during processing. Several images provided by institutions included picture frames or colour reference bars which were removed using Adobe Photoshop (adobe.com/uk). TIFF images were used to measure contrast and fractals and converted to JPEG to measure colour to meet software requirements.

The images were necessarily produced under various lighting conditions and using varying equipment which may have affected the results although there is reason to believe that this variation enhances overall reliability of results by reducing the likelihood of any one format biasing the data (189). The images had varying numbers of pixels which were not standardised as test results of all variables, contrast, colour and fractal dimension, using several resolutions, were found to be substantially the same after rounding.

### Statistical approach

The principal empirical approach to the data is correlative. That is, to determine whether changes in the variables, contrast, colour and fractal dimension, as well as productivity, correspond to critical events/periods in Rembrandt’s life and if so in what way and whether such changes are consistent with potential psychopathology. As only self-portraits and portraits, as opposed to the entire oeuvre, were examined the sample size is relatively small. The data points do not form a normal distribution and are dispersed and incomplete. These factors ruled out the use of common parametrical statistical tests such as ANOVA. To deal with these inherent problems non-parametrical statistical tests, Median and Mann-Whitney, were applied. The former tests whether the two medians (each variable in non-critical and critical years) are the same. Mann-Whitney tests whether the two distributions are the same. The Wilcoxon test was applied to test for changes in intervals including both non-critical and critical years. Thus, the principal focus was to compare the central values of each tested variable in non-critical and critical periods.

Non-parametrical tests generally do not have the same statistical power as those applicable to normal distributions (190). They also are sensitive to sample size, data completeness and dispersion. The number of statistical tests applied may suggest the use of multiple comparisons corrections to avoid the finding of spurious results. However proper application of multiple comparisons procedures such as the Bonferroni correction becomes too conservative if the tests are correlated amongst each other both because the correlation is endogenous to the paintings (e.g. as in the case of application to different colour channels), or because it is exogenous (e.g. self-portraits and portraits are made by the same artist). Hence these corrections were not applied. This may result in a very small number of false positives if any.

### Contrast

One study of Rembrandt’s self-portraits using digital techniques to measure luminosity detected different use of chiaroscuro, a form of contrast, in his early and late as opposed to his middle period (191). To measure changes in levels of contrast in this study an average measure of contrast was derived from each painting. In digital images the luminance of a pixel is derived from its red, green, and blue (RGB) wavelength components as 0.299R + 0.587G + 0.114B. The images were analysed as a two-dimensional random process and the mean and autocorrelation function provided a value for the contrast of brightness. The autocorrelation function quantifies the average relationship between data points in a time series and their previous data points using the Wiener–Kinchin theorem (192). Matrix transformation of luminance values was conducted using the 2-D Fourier Fast Transform and the average spatial contrast was characterized by the value at origin (184). The calculations were performed using MATLAB (v. R2021a).

### Colour

Examination of the use of colour in art using digital images and statistical techniques has yielded some insights. A large study of paintings from the 15th to the 20th century identified levels of colour contrast associated with particular artistic styles (193). A computational analysis of colour range was used to distinguish Renaissance from Baroque painters noting a decline from the earlier to the later period (194). Fractal colour analysis has been used to track both changes in colour and the use of chiaroscuro from the medieval to the modern era (195). More recently it has been used to track the relationship of colour and complexity with emotional and physical pain (185).

Here for each painting the colour median was measured in Red Green Blue (RGB) and Cyan Magenta Yellow Black (CYMK) models. RGB, often known as additive colour mixing, is a method of encoding the three cone receptor wavelengths. In concept, each colour is made up of red, green, and blue light that shines with varying intensities. Hexadecimal coding allows integers ranging from 0 to 255 to be represented with only two digits. The CMYK colour model is a subtractive colour model used in colour printing. It subtracts or masks colours from the white backdrop of the paper as ink decreases reflected light. The RGB and CMYK average values were calculated using MATLAB (v. R2021a) which applies consistent ranges of intensity in greyscale images to identify the specified colours.^4^

### Fractal dimension

As mentioned above, fractal dimension has been used to detect stylistic changes in artist’s work related to altered behaviour such as alcohol consumption (151, 169, 170, 184) and to neurological disease (105). There are two common methods of fractal analysis: one ‘2’ and one ‘3’ dimensional. Both utilise “box counting” a widely used sampling and data collection process. The fundamental approach is converting the image to greyscale and placing a sequence of grids of decreasing calibre (boxes) over a picture methodically and recording data for each subsequent calibre (counting) creating grids of progressively larger sizes. The 3 dimensional method has been selected here as considerable colour information is lost after conversion to greyscale which can affect the accuracy in roughness measurement.

Every pixel of grayscale images has only one intensity channel; pixels of colour images have multiple intensity channels made up of red, green and blue. In order to reduce potential inaccuracy, a modified differential box counting approach (197) was used to determine fractal dimension values of RGB colour images. Combining the “probabilistic algorithm” method (198) and the “box merging” method extending box counting, (199) the fractal dimension of the resulting “3D” colour images was estimated using vectors in 5-dimensions (x, y, r, g, b). A greyscale image in this format generates 256 distinct shades of colour between 0 and 255. Applying this method to the 24-bit format for colour images resulted in individual RGB components calculated based on six possible combinations ranging from I_x,y_ (1) to I_x,y_ (6) which are averaged to improve accuracy of the roughness estimate. The range of D values using this method is between 2 and 3 which were plotted with log(S) on the x-axis and log(N(s)) on the y-axis. The midpoint of 3D analysis, 2.5, is equivalent to 1.5 in 2D analysis.

## Results

If Rembrandt was depressed one would expect evidence of altered levels of contrast in paintings completed during Critical Years. His perception of both brightness and colour in his environment would diminish and contrast reduced or heightened to compensate. Depression might also be evidenced by a shift toward brighter background colours such as yellow. A shift towards yellow in old age would also be consistent with age-related macular degeneration. Reduced fractal dimension, complexity, would be consistent with depression, cognitive decline, or neurological disorder. A further indication of depression would be reduced productivity.

### Contrast

**Figure 1:**
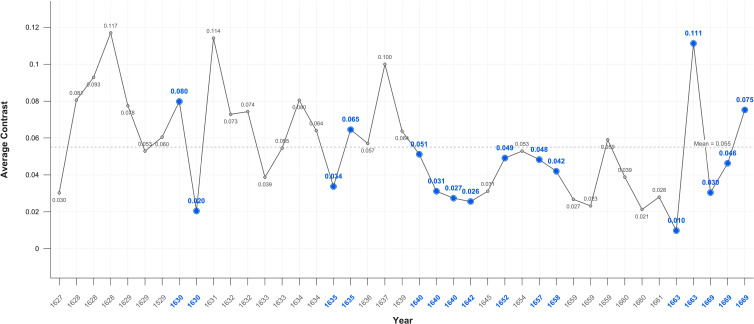
Average contrast– self portraits.

**Figure 2:**
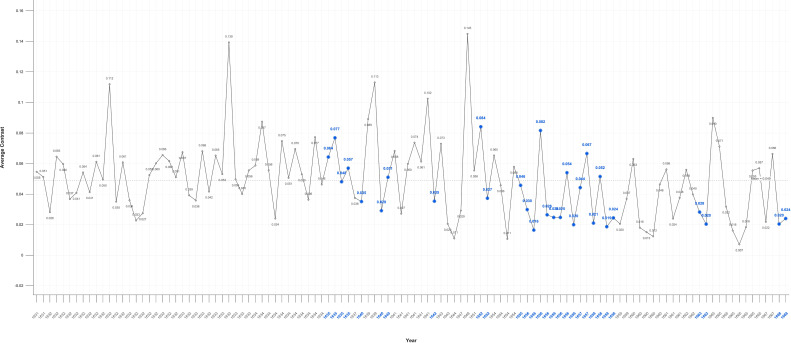
Average contrast – portraits.

The level of average contrast in Rembrandt’s self-portraits, varies for the first half of his career but falls in 1640, the year of his mother’s and a third infant’s death and two years before his wife’s death; it remains below average for the following 23 years. It is noteworthy that it increases close to the mean during his bankruptcy and again in 1663, the year of his mistresses’ death (**Figure 1**). This is consistent with a previous study of his use of chiaroscuro (191). The self-portrait on which he worked throughout his late period (1663-1669) has a markedly lighter background. The mean levels of average contrast in his self-portraits (0.55) and portraits (0.53) are relatively stable but both are noticeably lower during Critical Years; 0.47 and 0.40 respectively (**Figures 1**, **2**).

**Figure 3:**
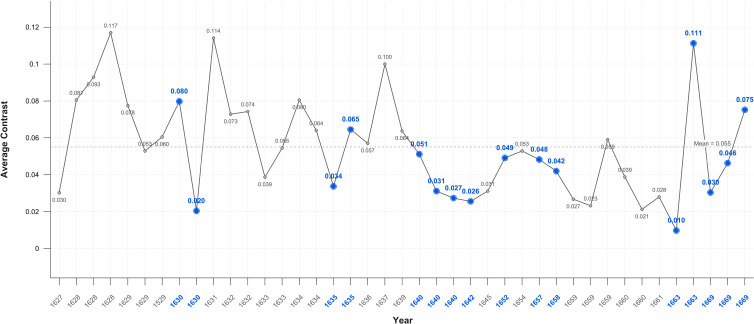
Average contrast portraits – family and friends.

**Figure 4:**
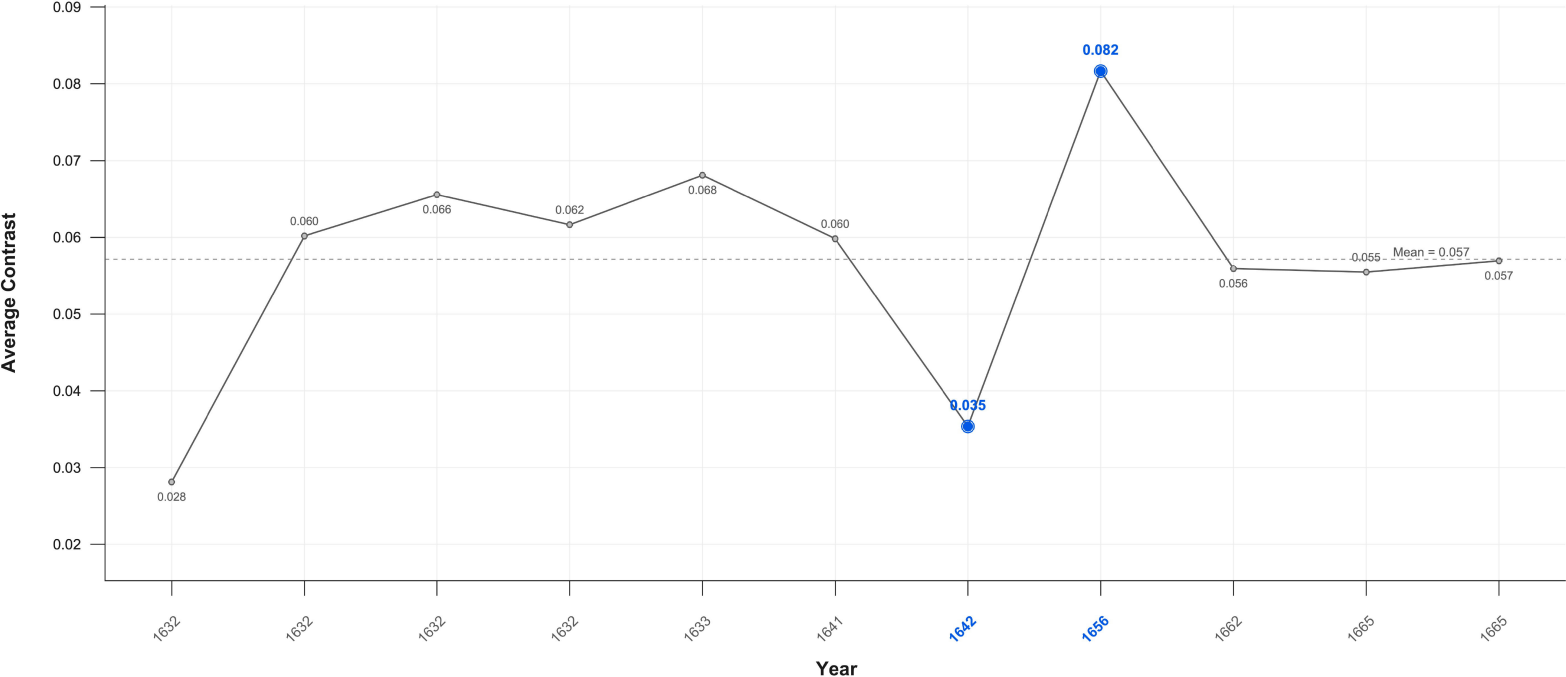
Average contrast – group portraits.

**Figure 5:**
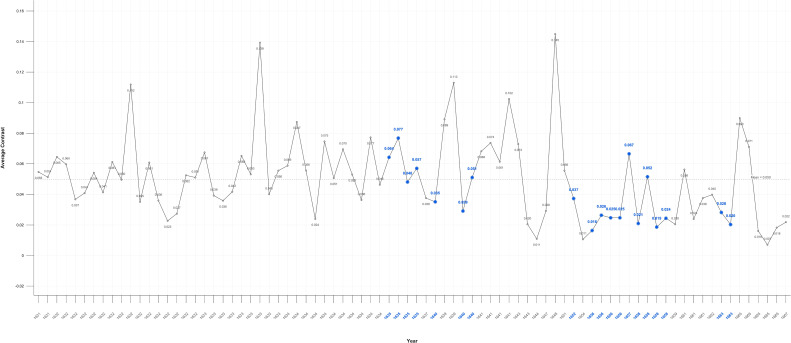
Average contrast – Portraits excluding groups and family and friends.

When the portraits are disaggregated the evidence is mixed. In Critical Years there is no meaningful decline in portraits of family and friends, in group portraits the mean level of contrast rises and the level of contrast of individual portraits of individual unrelated sitters is markedly lower. There are both increases and decreases in contrast in Critical periods in all three categories (**Figures 3**–**5**).

**Figure 6:**
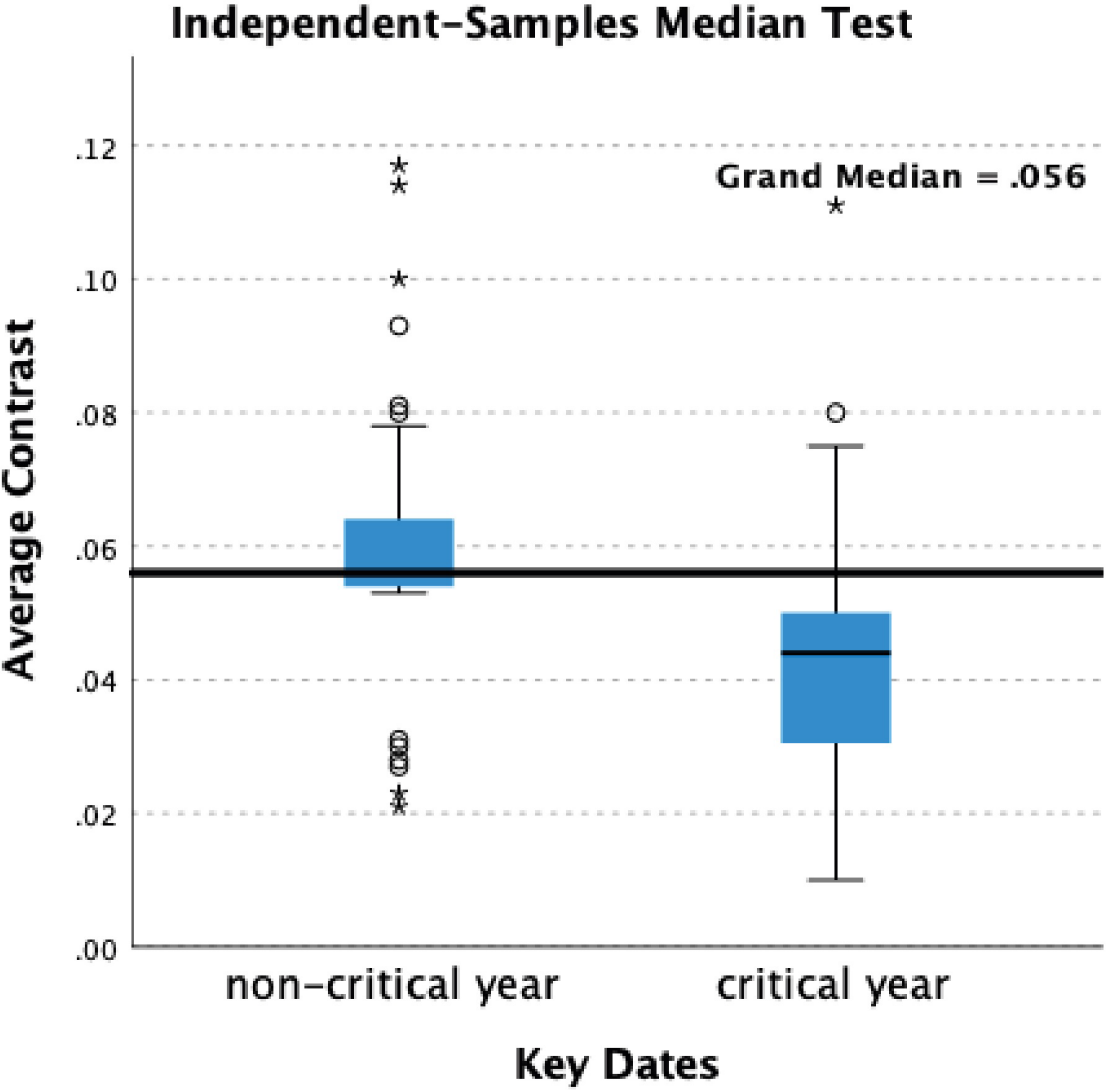
Box-plots – average contrast – self-portraits.

**Figure 7:**
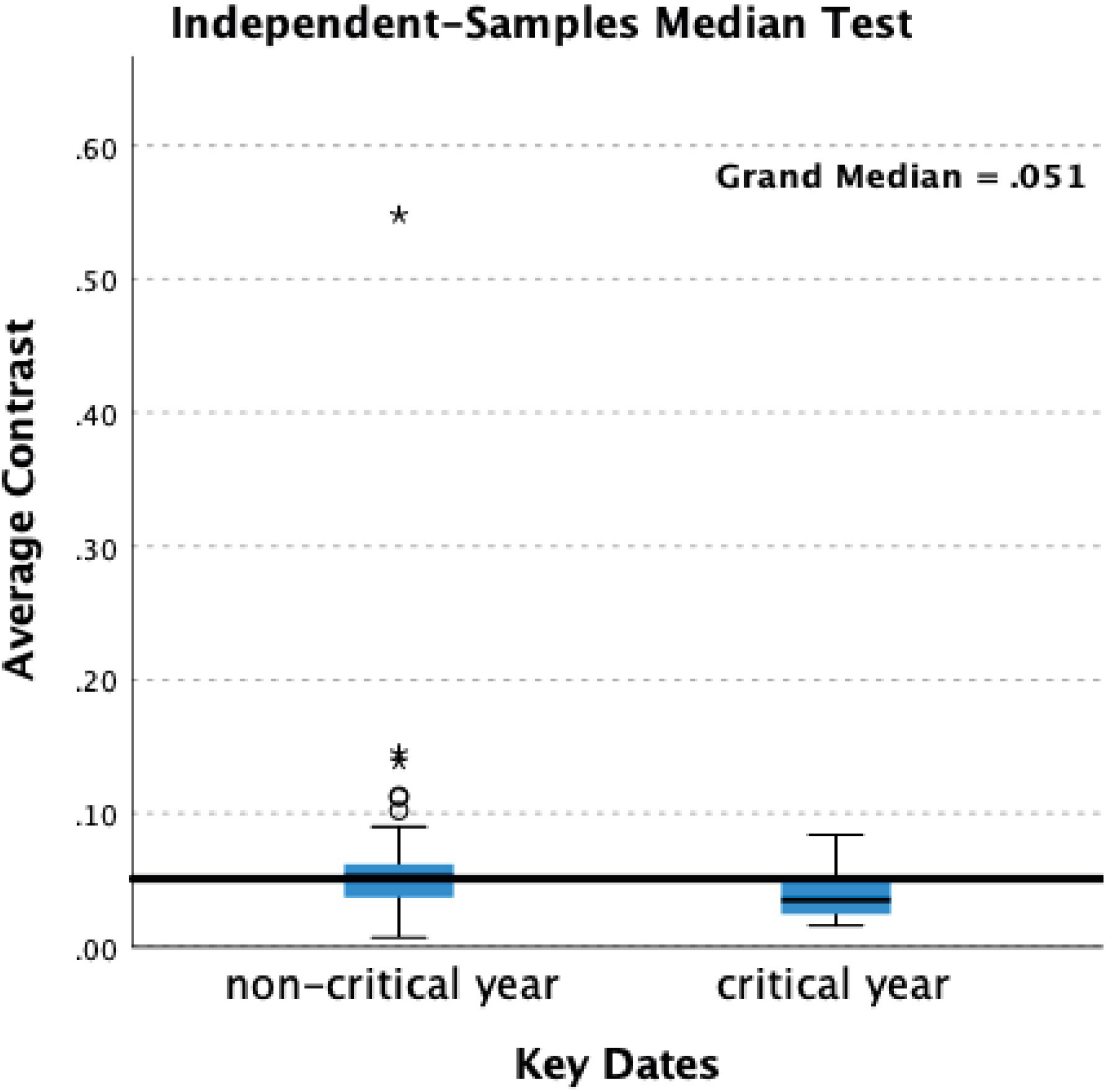
Box-plots – average contrast – portraits.

Statistical analysis indicates significant differences in contrast. In the self-portraits when Critical and non-Critical Years are compared there is a significant difference (p=0.028) in the Median test result. In the portraits there are significant differences in both Median (p=0.021) and Mann-Whitney (p=0.017) test results (**Table 1**). The decline in brightness in Critical Years is, however, noticeably greater in the self-portraits compared with the portraits (**Figures 6**, **7**).

**Table 1:** Average contrast*.

Group	R	GM	NCM	CM	MT P	M-W P
SP Contrast	0.010-0.117	0.053	0.56	0.044	0.028	0.092
P Contrast	0.007-0.548	0.05	0.053	0.035	0.021	0.017

*SP, Self-portraits; P, Portraits; R, Range; GM, Grand Median; NCM, Non-Critical Median; CM, Critical Median; MT P; Median Test p-value; M-W P; Mann-Whitney p-value).

### Colour

Primary colours were used sparingly by Rembrandt who virtually eliminated blue and green from his palette early in his career, before any adverse life event. Thereafter his palette was dominated by white, black, earth tones, brown, yellow and red. He continued to use smalt, a cobalt glass, to accelerate drying and thicken the texture of paint layers but not as a pigment. Unlike his other colours, which are stable, smalt fades to grey/brown over time thus analysis may underestimate the amount of blue originally present (188, 200).

**Figure 8:**
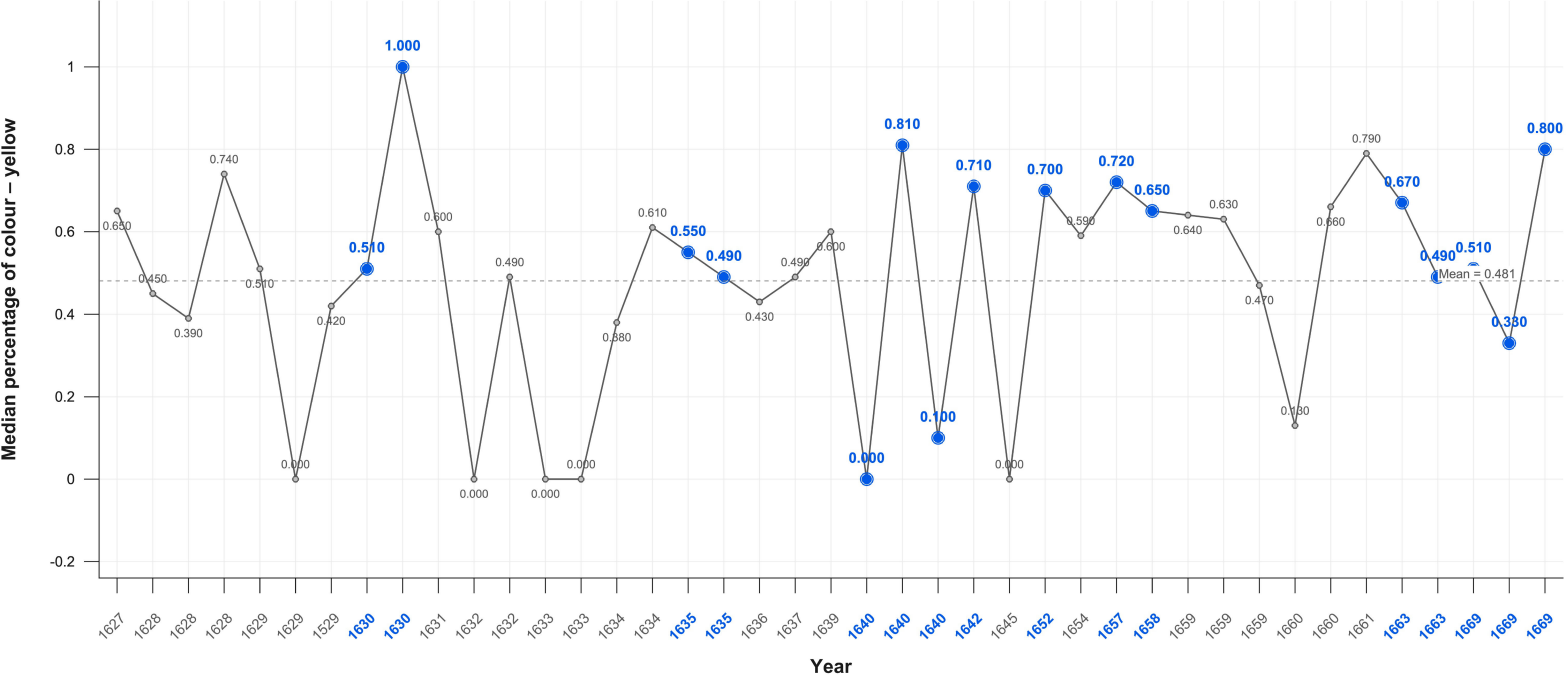
Median percentage of colour – yellow self portraits.

**Figure 9:**
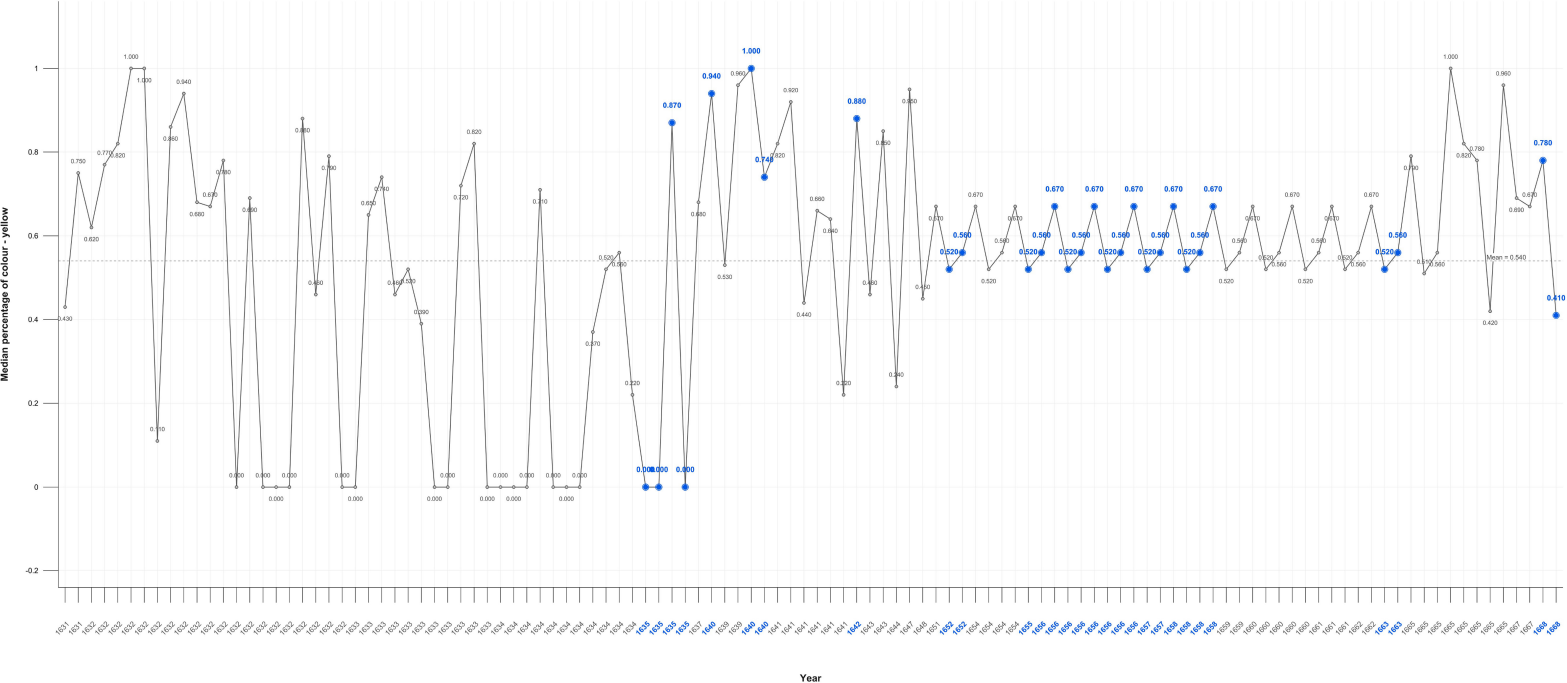
Median percentage of colour yellow – portraits.

When Rembrandt’s use of all colours is analysed there is a fall overall after his wife’s death and an overall rise during his bankruptcy followed by a surge in the year of his mistress’s death. There are comparable variations in the absence of adverse life events which together indicate a lack of correlation. When blue is isolated there also appears to be no meaningful difference in its use in Critical Years. The mean level of yellow, however, increases in both self-portraits and portraits in Critical Years (**Figures 8**, **9**). There is a noticeable reduction in the use of blue beginning in 1657 and an increase in yellow beginning in 1660. Statistically the only significant differences are in the use of magenta and yellow in the self-portraits as measured by the Mann-Whitney test. There are no significant results in the tests of portraits (**Table 2**).

**Table 2:** Colour*.

Group	Colour	R	GM	N-CM	CM	MT P	M-W P
SP	Red	20-85	43	42	42	0.845	0.822
SP	Green	13-74	37	34	36.5	0.956	0.968
SP	Blue	2-56	26	24	27.5	0.181	0.606
SP	Cyan	0-1	0.52	0.575	0.545	0.656	0.188
SP	Magenta	0-1	0.52	0.56	0.59	0.341	0.055
SP	Yellow	0-1	0.51	0.595	0.6	0.486	0.055
P	Red	2-188	32	32	32	0.871	0.608
P	Green	2-197	27	27	27	0.631	0.887
P	Blue	4-199	22	22	21	0.96	0.664
P	Cyan	0-1	0.65	0.595	0.73	0.307	0.147
P	Magenta	0-1	0.67	0.61	0.73	0.179	0.15
P	Yellow	0-1	0.67	0.63	0.75	0.177	0.148

*SP, Self-portrait; R, Range; GM, Grand Median; NCM, Non-Critical Median; CM, Critical Median; M TP, Median,Test p-value; M-W P, Mann-Whitney p-value; P, Portraits.

### Fractals

**Figure 10:**
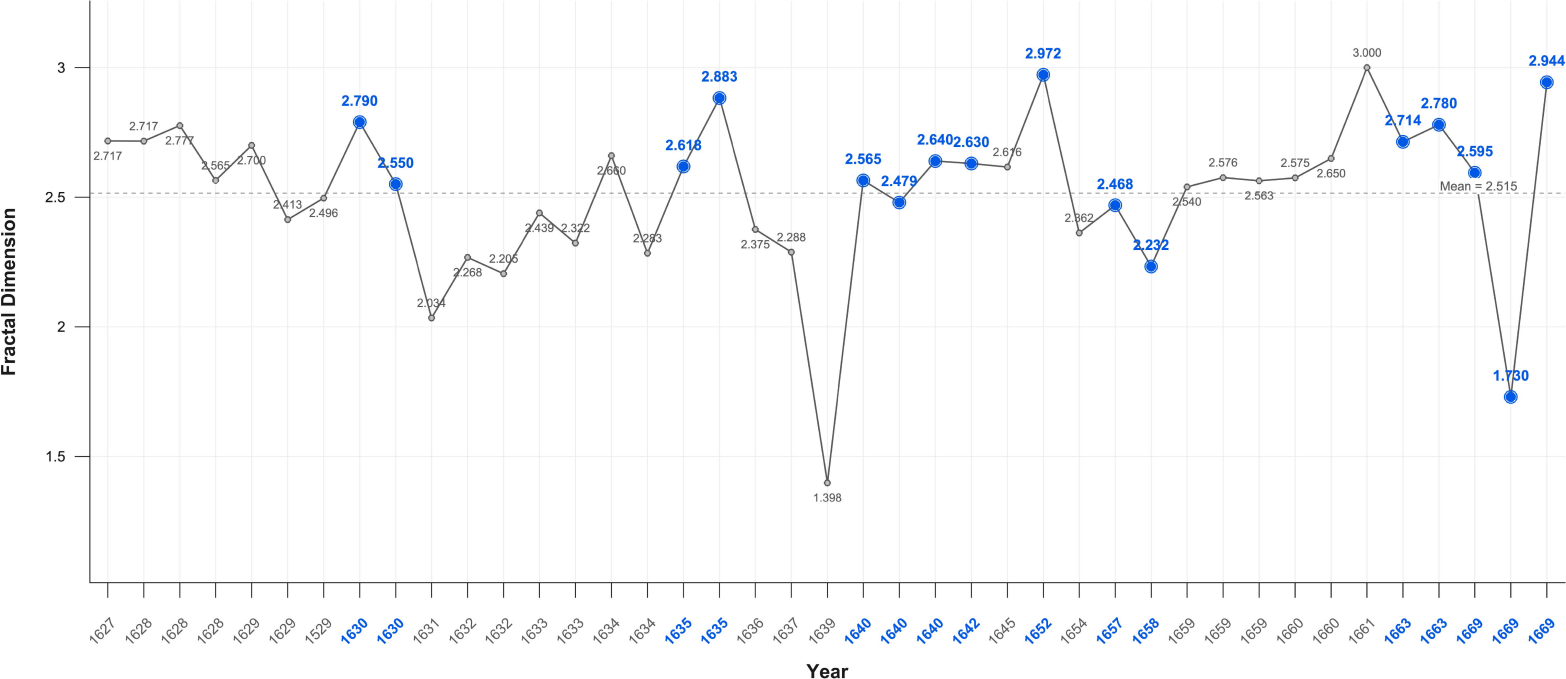
Self-portraits fractal dimension.

**Figure 11:**
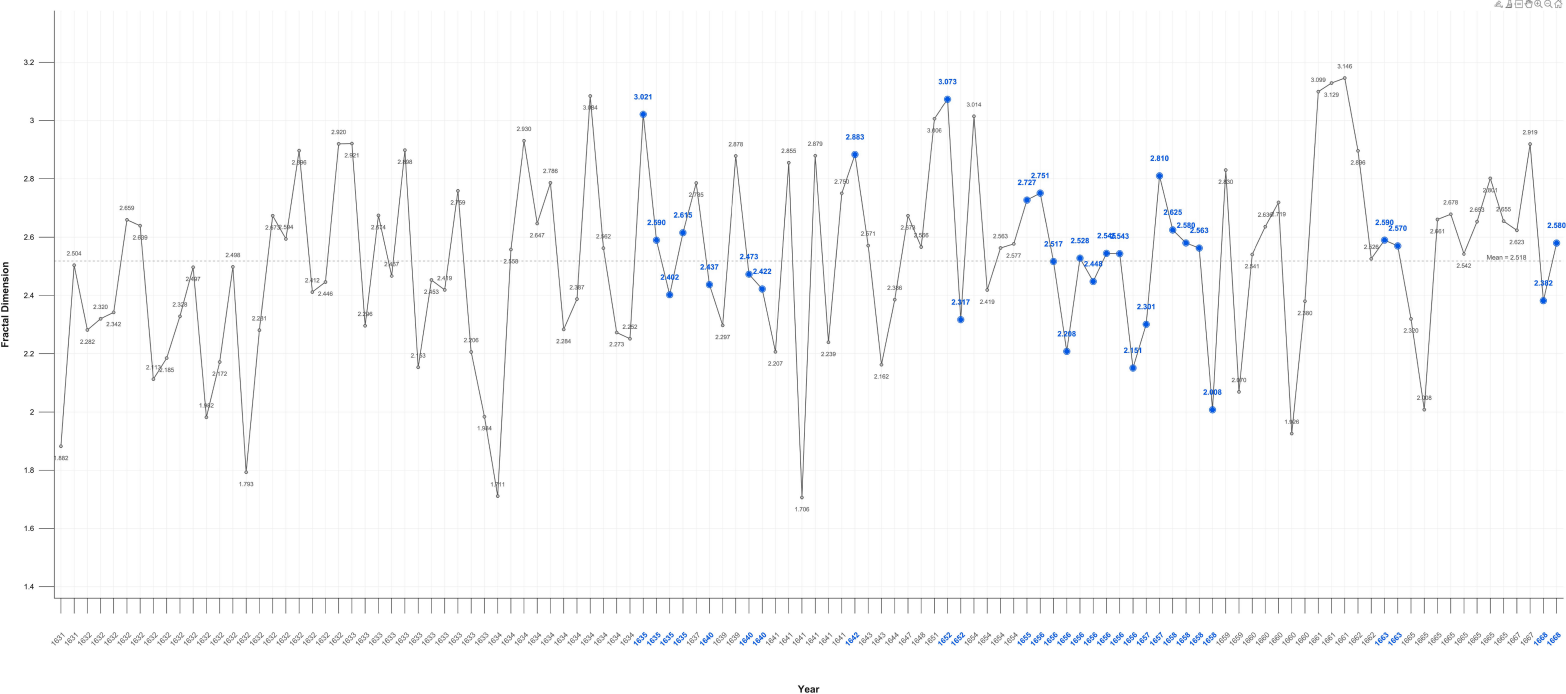
Portraits fractal dimension.

Fractal analysis proved sensitive in detecting significant variations between the self-portraits and portraits. While fractal values overall fluctuate near the mid-point of 2.5. there is an increase in mean values in Critical Years in the self-portraits and a decrease the portraits (**Figures 10**, **11**).

**Figure 12:**
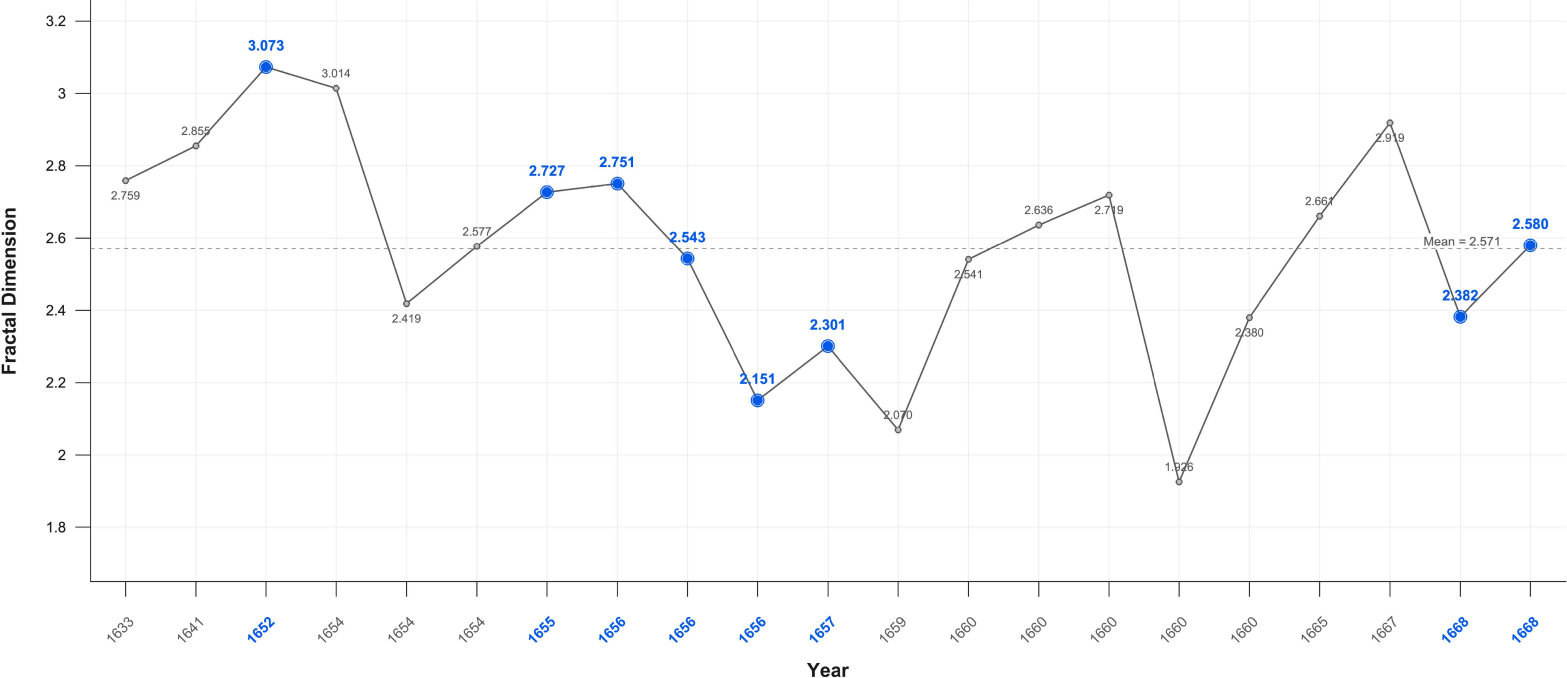
Portraits-fractal dimension: family and friends.

**Figure 13:**
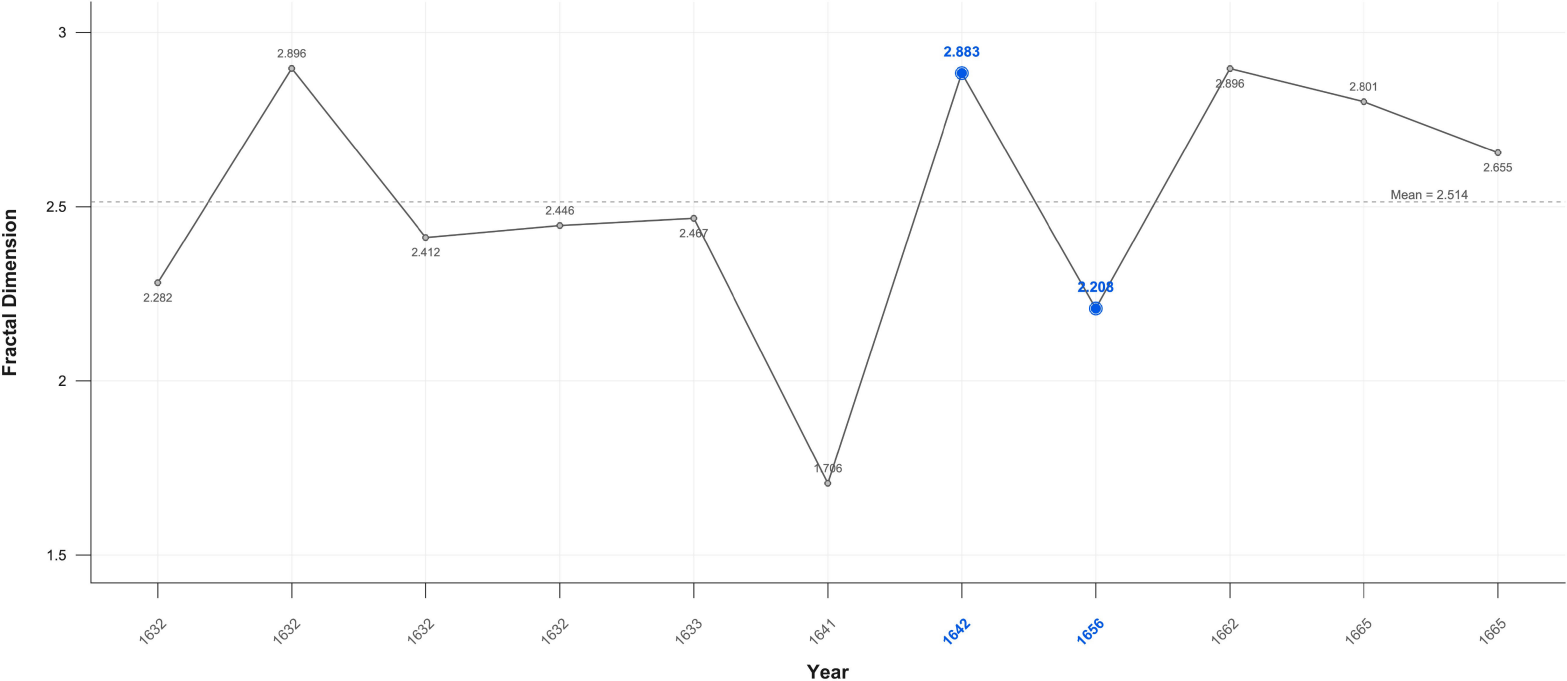
Portraits-fractal dimension: groups.

**Figure 14:**
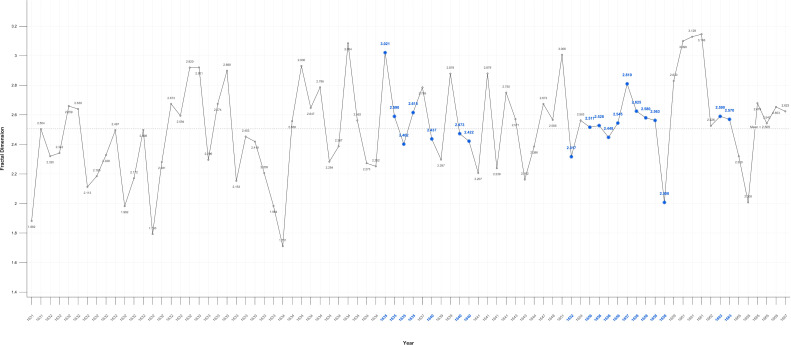
Portraits-fractal dimension excluding groups and family and friends.

When disaggregated related portraits have noticeable higher 2.571, and unrelated portraits noticeably lower 2.509 mean values as compared to portraits generally: 2.52. In Critical Years the D value rises above the mean in all categories of portraits, except related individuals (**Figures 12**–**14**).

There is a large, near significant, increase in mean fractal dimension in Critical Years in the self-portraits but very little change in the portraits (**Table 3**).

**Table 3:** Fractal values*.

Group	Measure	R	GM	N-CM	CM	MT P	M-W P
SP	Fractal 3D	1.398-3.000	2.565	2.394	2.824	0.16	0.078
P	Fractal 3D	1.706-3.146	2.545	2.55	2.545	0.872	0.882

*SP, Self-portrait; R, Range; GM, Grand Median; NCM, Non-Critical Median; CM, Critical Median; MT P, Median Test p-value; M-W P, Mann-Whitney p-value; P, Portraits.

This variation becomes clearer when Critical periods are examined. There is a statistically significant increase in D in self-portraits produced near Critical dates (Mean FD = 2.650 Std = 0.174) compared with those produced during non-critical periods (Mean FD = 2.453 Std = 0.338); Wilcoxon test p=0.05. On or near Critical Years there is an increase in complexity in the self-portraits but there is no significant difference in the portraits between non-Critical periods (mean FD = 2.501 Std=0.332) and those on or near Critical dates (mean FD = 2.566 Std = 0.245).

### Productivity

**Figure 15:**
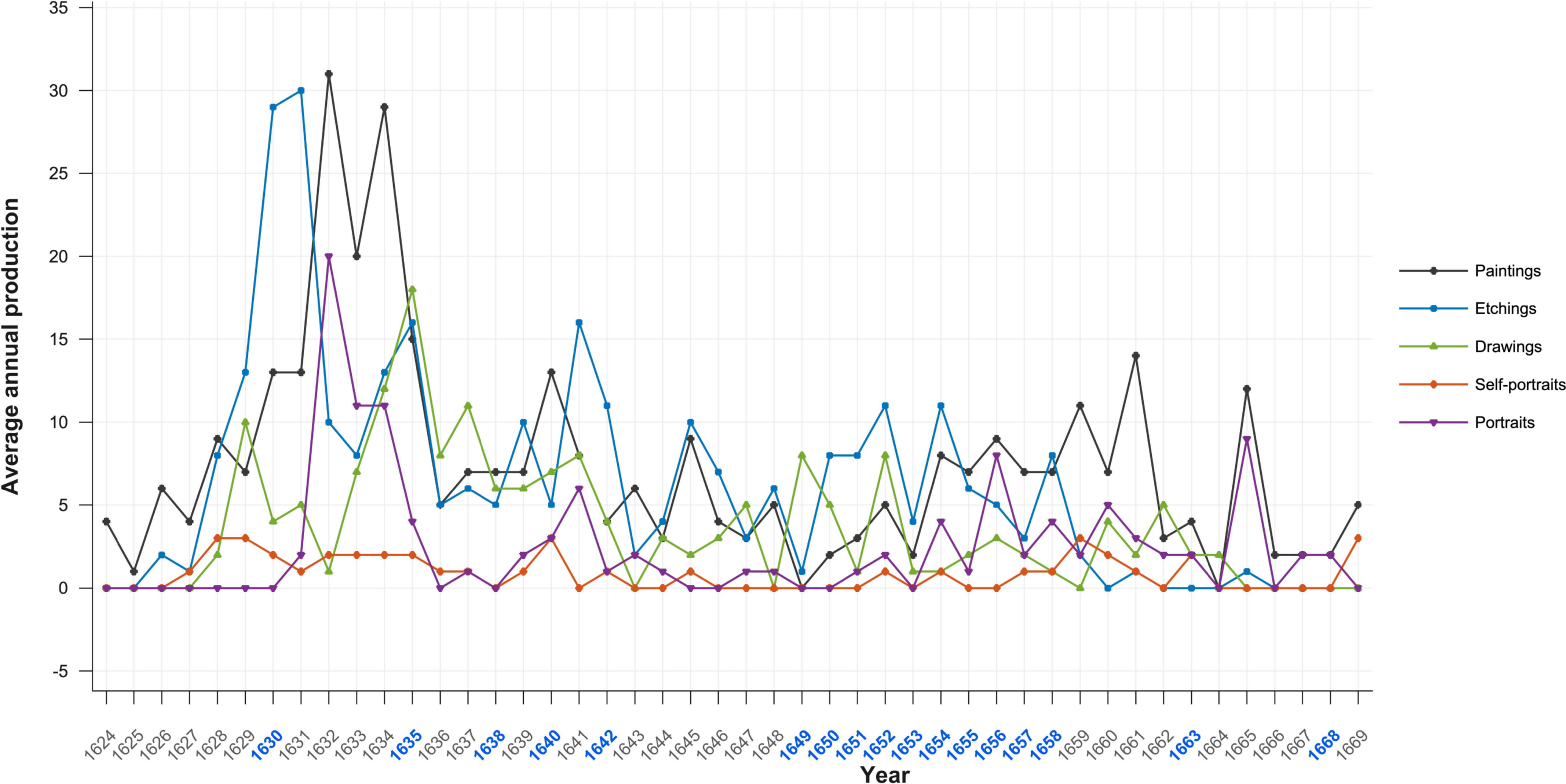
Average annual production – paintings, etchings, drawings, self-portraits & portraits.

**Figure 16:**
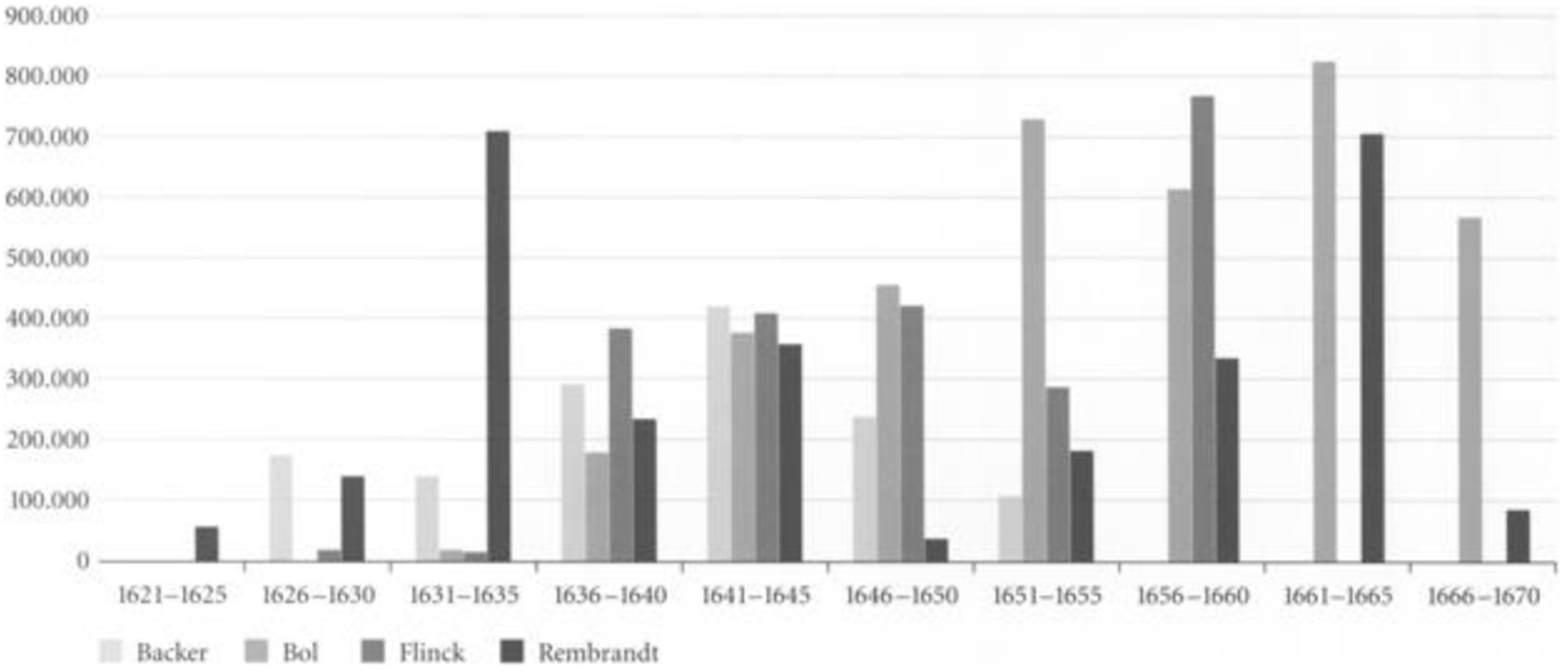
Total surface area of paintings by Backer, Bol, Flinck and Rembrandt in five-year periods (Y=cm^2^) (201) reprinted with permission.

Productivity in artists can be measured both quantitatively and qualitatively. When measured quantitatively, with the exception of the earliest, Rembrandt’s productivity increases in Critical periods. His annual productivity measured in terms of number of paintings, etchings, drawings, self-portraits and portraits is illustrated in **Figure 15**. Others have measured his productivity in terms of surface area painted (**Figure 16**). Although there is no statistically significant relationship between volumes of categories of work and Critical and non-Critical years, in all but one category, self-portraits where it is equal, mean volume is higher in Critical Years in all categories (**Table 4**).

**Table 4:** Annual productivity – volume*.

Type of work	R	GM	NCM	CM	MT P	M-W P
Paintings	0-31	6.5	6	7	1	0.656
Etchings	0-30	5	3	6	0.65	0.155
Drawings	0-18	2	2	3	0.821	0.284
Self-portraits	0-3	1	1	1	0.964	0.864
Portraits	0-20	1	1	2	0.821	0.672

*SP, Self-portrait; R, Range; GM, Grand Median; NCM, Non-Critical Median; CM, Critical Median; MT P, Median Test p-value; M-W P, Mann-Whitney p-value; P, Portraits.

When measured in terms of area painted Rembrandt’s productivity is consistent with volume measurements. The area painted fell for the first time in the first period of adverse events in his life (1636-1640). In the following Critical period (1641-1645) the time of his wife’s illness and death, the area he painted increased by two-thirds; from 225,000 to 375,000 square centimetres. In the subsequent non-Critical period (1646–1649) productivity measured by number and area painted fell below their long term average. From a low point in 1650 his output increased until it reached its long-term average of 320,000 square centimetres in 1660; that is, it increased significantly during his bankruptcy. In the five year period (1656-1660), which includes the bankruptcy’s culmination his productivity continued to rise. In the following five year period (1661-65) Rembrandt’s productivity reached a peak of 700,000 square centimetres equalling the level reached in the first half of the 1630s (**Figure 16**).

Conclusions drawn as to productivity from quantitative measures must, however, be qualified as we do not know how many paintings and drawings have been lost nor how many prints were made (202). For example, in 1641, when the number of his paintings appears to decline, we know that he painted an Ovidian series of paintings now lost. We know neither how many nor what size they were (20). If included the numerical decline in that year might be reversed and the area painted increase. Paintings also continue to be discovered.^^5^^ Thus, it is likely that current quantitative data underestimate his productivity.

## Discussion

The results indicate that Rembrandt’s self-portraits evidence low mood, but not depression, in response to adversity. In the self-portraits Critical years are significantly correlated with reductions in contrast, increases in the use of yellow and higher fractal dimension. Analysis of the portraits shows a significant, but mixed, decline in contrast, no change in colour and no significant increase in complexity. Comparison of the results indicate that the self-portraits are, as hypothesised, distinct in style from the portraits and suggest that they reveal Rembrandt’s state of mind to a greater extent consistent with their higher saliency. There also appears to be a gradient in Critical periods in fractal dimension. The highest values in the self-portraits, followed by related portraits and then unrelated portraits are consistent with experimental, anatomical and functional analysis of image saliency. The evidence of increasing productivity in Critical periods is consistent with resilience in the face of adverse events. This and the consistent mid-range fractal levels in both self-portraits and portraits, together with the absence of any decline in fractal values in old age, are in accord with the hypothesis of neurologically and cognitively healthy aging. There is also evidence, consistent with the hypothesis of normal aging and connoisseurship, of age-related macular degeneration, a decrease in blue and an increase in yellow, as well as increased contrast, in his last years.

### Contrast

The development of artistic techniques, including chiaroscuro, used extensively by Rembrandt, has been previously tracked by detecting changes in brightness (195). An effort to analyse the changes in chiaroscuro in the faces of several of Rembrandt’s self-portraits, although limited in scope, distinguished originals from copies and revealed that his use of this technique was similar in his early and late periods but quite different in middle age (191). The view of most art historians is that the change was the result of deliberate changes of style in 1640 and again in 1663 (6). The late rise in contrast is consistent with macular degeneration and a perceived shift towards yellow (38; and see Colour below).

The current study shows a reduction in average contrast in his self-portraits that began before his wife’s death and lasted for more than two decades, including his bankruptcy, then increasing once more. There is also a decline in contrast in the portraits when measured in aggregate. The duration of the decline in contrast could thus indicate dysthymic depression, however the level of contrast from the portraits when disaggregated shows a rise in contrast in the Group portraits and no change in those of Family and Friends. This is inconsistent with an overall darkening of perception. The decline in contrast in the portraits thus appears limited to unrelated individual portraits consistent with a deliberately chosen style for such works.

The significant correlation between Critical Years and lower contrast in the self-portraits may be indicative of low mood albeit that the periods of lower contrast are typically followed by increases in contrast in non-critical years (**Figure 1**). This suggests a transient darkening of mood followed by recovery typical of healthy response to adverse events. In the self-portraits when Critical and non-Critical Years are compared there is a significant difference (p=0.028) in the Median test result. In the portraits too there are significant differences in both Median (p=0.021) and Mann-Whitney (p=0.017) test results (**Table 1**) however the decline in brightness in Critical Years is noticeably greater in the self-portraits as compared with the portraits (**Figures 6**, **7**). This suggests that Rembrandt’s reaction to adversity is particularly expressed in the self-portraits.

### Colour

Rembrandt’s use of colour, his palette, is known to have changed, removing blue and green, early in his career and before any adverse events. The results do not indicate any significant correlation between use of colour in Critical and Non-Critical periods other than yellow and magenta (Mann-Whitney p=0.055) in the self-portraits (**Table 2**). The mean level of yellow increases in both self-portraits and portraits in Critical Years (**Figures 8**, **9**). The increases in the use of yellow in Critical periods is consistent with an effort to compensate for a sense of dimness, reduced contrast, or lack of perceived colour in his environment in such years.

There is a noticeable, but not statistically significant, reduction in the use of blue beginning in 1657 and an increase in yellow beginning in 1660 consistent with age-related macular degeneration and/or diminished perception due to depression. This is consistent with connoisseurs who observe a ‘yellow-brown’ shift in the last self-portraits three of which were painted and one finished in his last year (6, 38). There is, however, no apparent loss of visual acuity or blurred vision attested to by the extraordinary detail in his late self-portraits which led to various diagnoses.

Except for yellow-blue, colour does not appear to be associated with Critical periods. There is an overall decrease in the use of colour after his wife’s death, but there are increases during his bankruptcy and in the year of his mistress’s death. There are comparable variations in the absence of adverse life events. The colour variations are also of short duration, with sudden rises and falls, inconsistent with observations that the world overall has less colour in depression. This suggests that the significant increases in yellow in the self-portraits served to compensate for reduced contrast at times of adversity and later for macular dysfunction.

### Fractals

The fractal mean value of the self-portraits and all categories of portraits varies only marginally from 2.5 which is precisely at the ‘cusp,’ of the widely recognised mid-range of natural and aesthetic preference. This is the point at which arousal, attention and beauty reach the optimal level before preference declines (105, 151, 203). There is nevertheless a statistically significant increase in self-portraits produced near Critical dates compared with those produced during non-Critical periods. There is no significant difference in the portraits between non-critical and those on or near critical dates. This suggests, consistent with the contrast and colour results, that self-portraits are more sensitive to changes in mental states.

The increase in complexity in the self-portraits on or near Critical Years is consistent with EEG findings in MDD showing moderate increases in fractal dimension, complexity, in both beta and gamma wave analysis of frontal and parietal areas (175, 204). Nevertheless, even these elevated D values remain with the range maintained throughout Rembrandt’s career indicating transient changes in mood and militating against a diagnosis of persistent unipolar or bipolar depression. There is also no correlation with age, so the effect was temporary.

The higher self-portrait D values in Critical Years and Periods support the hypothesis that self-portraits are distinct from and a subject of more intense focus than images of others. The gradation in Critical years’ mean fractal values from the self-portraits (2.599), to related portraits (2.564), to unrelated portraits (2.529). is consistent with the experimental evidence of a saliency gradient of self-images, images related to the self, and unrelated images. This is functionally and anatomically reflected in a ventral-dorsal gradient in the mPFC (205–208).

The higher D values in Critical Years and during Critical Periods also indicate an increase in larger spatial frequencies as compared with lower spatial frequencies. This may be due to changes in the backgrounds in the paintings. Visual comparison of self-portraits in Critical and non-Critical periods does not reveal obvious differences. This may be an effect of the wide variety of materials and techniques Rembrandt used in backgrounds some of which are still being discovered (188, 200, 209). Many of Rembrandt’s effects are the result of alterations of the surface by brushstroke, paintbrush tip, palette knife and other three dimensional techniques not measured here.

### Aging and productivity

Artists with Alzheimer’s. Parkinson’s and Lewy Body disease show significant declines in the fractal dimension as disease progresses. Their art is characterised by reduced complexity, less detail and greater abstraction (210). Analysis shows no such decline in Rembrandt’s self-portraits or portraits. The fractal levels in old age are consistent with those of early and mid-adulthood which appears to exclude neurogenerative disease or cognitive deterioration due to aging. There is no evidence of the other cognitive or motor symptoms of degenerative disease. This suggests that he did not suffer from age-related cognitive decline and had considerable cognitive reserve (211). He continued to paint until his death leaving behind both completed and unfinished works (22, 212).

Although there is no statistically significant relationship between volumes of categories of work and Critical and non-Critical years, in all but one category, self-portraits where it is equal, volume is higher in Critical Years. After his wife’s death the number of his paintings rose, the number etchings and drawings fell then all three returned to their average levels until the beginning of his financial difficulties in 1649 when all fell in number, likely contributing to those problems. From that point on until 1653 the number of paintings and etchings, from which, unlike drawings, he derived significant income, rose (2, 20, 202, 213, 214). While both fell in 1653 they thereafter continued to rise in number through and beyond his bankruptcy. It is evident that Rembrandt’s productivity, whether measured in number of paintings, etchings or drawings, individually and in combination, was sustained throughout his career albeit reduced three years before his death when it began to increase once more. Importantly it increased, in all categories, during Critical Periods and decreased in non-Critical Periods.

In the period 1636–1640 he lost three children and his mother. His productivity measured by surface area painted dropped sharply compared with the previous five years, however, this also marks the period following his leaving the workshop of Hendrick Uylenburgh which had provided a steady stream of commissioned portraits. The drop in volume thus likely reflects the time it took for him to develop a clientele independently (2, 187). In the following the five year period (1641-1645) which includes the last year of his wife’s illness, her death and the three years following, the area he painted increased over the previous period by two-thirds; from 225,000 to 375,000 square centimetres. Between 1646 and 1649, a non-Critical period, Rembrandt’s productivity measured by number and area painted and number of etchings fell below their long term average. This was based on his decision to cease painting portraits on commission, except when in need of money, and to concentrate on more prestigious and costly history paintings which contributed to his financial distress when they failed to sell (2, 20, 201).

As a result of his decision his income fell and in 1649 he failed to make a mortgage payment which was the principal event leading to bankruptcy. From that point on his productivity in number of paintings and etchings and surface area painted began to increase and continued to do so for the next 15 years. From a low point in 1650 his output increased until it reached its long-term average of 320,000 square centimetres in 1660; that is, it increased significantly during the period of his greatest financial stress. In the five year period (1656-1660), which includes the bankruptcy’s culmination including the sale of his house and art collection and move to modest rented accommodation, his productivity continued to rise. In the following five year period (1661-65) Rembrandt’s productivity reached a peak of 700,000 square centimetres equalling the level reached during his employment by Uylenburgh in the first half of the 1630s (**Figure 16**) (13, 201).

There is thus an inverse correlation between adverse life events and painted productivity; his productivity rising during his wife’s illness, death and in its aftermath and during and in the aftermath of his bankruptcy and declining in non-Critical periods. Although Rembrandt’s productivity, measured by surface area painted declined in the three years prior to the year of his death, measured by number of paintings it began to rise in his last two years and he left three completed and 26 unfinished paintings at his death (20, 22). It is therefore unsurprising that there are no statistically significant correlations between lower productivity and critical years or periods (**Table 4**). The number of self-portraits notably increased four being completed in the last year of his life. This is consistent with an increasing concern with his inner life and his legacy. His increase in productivity during and following his bankruptcy, can be seen as a rational and flexible response to events (2, 3, 20). In the aftermath of bankruptcy, in reduced circumstances, his productivity returned to the highest level in his career (201). This inverse correlation is counter-indicative of depression. One would expect productivity to fall in periods of illness or disorder other than mania which does not endure for periods measured in years. The evidence of productivity is therefore inconsistent with episodic, chronic/dysthymic or manic depression. It appears that adverse events, including old age, stimulated Rembrandt to work rather than despair,

Productivity in artists can be measured qualitatively as well as quantitatively. While no qualitative study was undertaken, the quantitative results are consistent with connoisseurship. His self-portraits, at the two most critical moments in his life, his wife’s death and his bankruptcy, events which may well have triggered depression in others, reveal resilience and self-confidence. In the year after his wife’s death he completed ‘The Night Watch’ perhaps his greatest and largest masterpiece and a self-portrait which depicts him in a confident pose modelled on Titian. In the year he lost his house and possessions he painted his only three-quarter length self-portrait in the posture of a prince and master of his profession. In the year following he portrays himself in the self-assured pose of a Renaissance courtier in the manner of Raphael. In his last four years he painted several of his finest portraits, including his only equestrian one, and several masterpieces including The Jewish Bride and The Return of the Prodigal Son (5, 7, 38). In his last self-portraits, painted in the four years before his death, he conveys life’s experience as no one has ever done before or since. There is a broad consensus among art historians and artists that Rembrandt’s late style was his finest.

His late creativity may have been a compensatory reaction to the losses he had suffered (215–217) but the paintings of this period show a master at the height of his power and using techniques which have been admired ever since by painters and connoisseurs alike (4, 5, 13, 218). His last self-portraits are remarkable technically as well as stylistically. Scientific examination of their materials reveal that Rembrandt continued to innovate until the end of his life (6, 29, 188, 200, 219). Thus, his productivity, measured qualitatively, does not appear to be related to adversity and can be seen to have increased in old age (13). His continued experimentation in technique and in many scholars’ view, continued growth as an artist, are inconsistent with depression, at least as clinically defined, and with cognitive decline due to age or neurodegenerative disease.

### Depression

Depression has been well summarised as the disproportionate experience of sadness and bereavement which do not remit when the external cause dissipates (220). The suggestion that Rembrandt suffered from depression has been based on various aspects of his life. Some have concluded that Rembrandt suffered from unipolar or bipolar depression based on the subject of two etchings, the decline in the number of etchings following the death of infant children and of his wife and his previous apparently extravagant use of his recently acquired wealth (11). Others have focused on his self-portraits analysing both objective indicators of disease and subjective assessment of mood 40. 41).

He was seen by his wife’s relatives and by observers at art auctions, as reckless with money and why he did not pay off his debts, particularly his large mortgage, when he had the means to do so remains perplexing (2, 20). Spending sprees are characteristic signs of mania and there is an association between mild symptoms of BPD, positive mood, and creativity (221). Rembrandt had elevated moments one of which he painted as a self-portrait with his wife (1635). Mania can manifest as intense work in short periods and heightened creativity was once regarded, in DSM-III (222), as a symptom of hypomania.

Looked at in isolation, the period shortly before and after his wife’s death might suggest mania followed by depression characteristic of BPD. It can also be regarded as elevated mood associated with healthy creative people or those who become wealthy for the first time followed by normal grief. It is noteworthy that the objects of his expenditure were on a house large enough to accommodate a family, a studio with up to 50 apprentices, an art dealership and a substantial collection of artworks and objects related to his work (200). There is no suggestion of abuse of alcohol, promiscuity, or other reckless behaviour characteristic of BPD. Mania would also not be characterized by the intervals of expenditure, measured in years rather than weeks or months, the career-long consistency of mid-level fractal values associated with health and beauty, nor the periods of steady growth in productivity including that of his wife’s illness and death.

Judging by the images he made of her he loved and felt the loss of his wife who he twice painted posthumously. However, there is an important distinction between healthy grieving (mourning) and pathological grief (melancholy) (223, 224). The evidence here suggests normal grieving and occasional periods of low mood due to adversity. After his wife’s death, while apparently grieving her loss he soon established intimate relations first with his son’s nurse and then an enduring relationship with his housekeeper. He also remained deeply attached to his son, and his engagement with students, friends, patrons and the art market never waned. There is no indication of dysfunction in any domain, personal or professional.

It is understandable that his last self-portraits are associated with the cumulative effects of the adverse events of his life. Although they were not unusual for a man of his time and place such experiences can have a cumulative effect (14, 15). To some they convey a sense of melancholy; this may be attributed to him or to the observers’ own reaction to his unsparing depiction of old age. The results of the analysis presented here suggests the latter is more likely. The etchings on which a diagnosis of unipolar and then bipolar depression were based (10, 11) may be of melancholy subjects but they are not images of Rembrandt. Whenever Rembrandt included his self-image in any work, he ensured that he was notably recognisable and never did so in a melancholy pose. None of the many contemporaries who mention him, including those critical of his work, ever so describe him although it was already fashionable for artists and poets to be ‘melancholy’. It is noteworthy that his first painted self-portrait and one of his last, depict him laughing (1628, 1663).

Rembrandt’s character has been described as eccentric, temperamental, disputatious even vindictive (3, 12). There is also agreement that he was a very hard-working, sober and dedicated artist capable of managing a large studio, dealing in art (his own and others’) and continuously developing his skills (2, 3, 187, 200). He did suffer both personal and professional setbacks but responded to them with increased effort unlike those suffering from depression. Rembrandt’s ability to do so may be explained by the number of protective factors which his life afforded him. There is no family history of depression or mental illness and his life experience included family cohesion, a stable home, good education and health, supportive friendships and social networks, high self-esteem, a sense of efficacy and of purpose, resilience and religious belief (225–229). Also notable is the absence of any reported childhood adverse experience. (230–232). The objective evidence here is consistent with health, both physical and psychological, throughout Rembrandt’s life.

Finally, some have suggested that Rembrandt’s refusal to follow changing artistic fashion later in life lead to less demand for his work and thus contributed to low mood. In fact, he remained in demand both in Holland and elsewhere (21). In 1661 he painted portraits commissioned by prominent Dutch patrons. In 1661–3 he completed two major commissions for an Italian patron and in 1666–67 he negotiated with another for a triptych (233). Cosimo de Medici visited him in 1667 and apparently purchased the self-portrait now in the Uffizi. Thus, although living in straightened circumstances he remained in demand and continued to cultivate the market for his art (234). There is no evidence of withdrawal or of becoming dysfunctional in any sphere of life (2, 12, 20, 235). There is, in short, no evidence of mania nor of disproportionate experience of sadness or bereavement and when he experienced such feelings they remitted when the cause dissipated and resulted in enhanced productivity and creativity including in old age.

## Conclusion

Informed commentators on Rembrandt’s self-portraits caution against projecting back modern ideas of self-expression onto 17^th^ century painters. Before the ages of the Enlightenment and Romanticism, artists were more likely to be motivated to paint their images to learn the art of expression or to advertise their name and skill. Indeed, the term ‘self-portrait’ did not exist at the time (236). There is reason, however, to argue that Reformation Holland was a crucible of the modern view of the individual and the increasing status of artists (4). In any event none of the various purposes which the self-portraits served precludes the expression of one’s state of mind. Rembrandt’s extraordinary capacity to depict facial emotion was noticed early in his career and has been appreciated ever since. It would be astonishing if he did not convey something of his feelings in his self-portraits. Few scholars doubt that the corpus of Rembrandt’s self-portraits, painted, etched, and drawn, was not only the first, but remains the most comprehensive, example in Western art of self-study if not autobiography (4, 5). This, however, does not indicate what his state of mind was when they were painted.

It is important, too, in considering the influence of mood or eye disease in artists not to fall into the ‘El Greco fallacy’ and confuse a chosen style with evidence of visual deficiency. Some concluded that El Greco’s figures were elongated because of astigmatism. Examination of his canvases not only showed that he initially drew the figures to scale and then elongated them when he painted, but experiment demonstrated that if astigmatism caused him to perceive his sitters as elongated, then the same astigmatism would self-correct the image when transferred onto canvas (237, 238). A similar error was made in a study of Rembrandt’s self-portraits which concluded from examination of the direction of gaze in each eye that he had strabismus (239). The authors overlooked the natural result of painting himself looking sideways at a mirror (2, 42). It is also evident that Rembrandt made conscious decisions to paint history paintings rather than portraits at a particular juncture and both those examining the self-portraits empirically and connoisseurs detect a change in style in his middle period. However, none of these studies nor any conscious stylistic choice, can explain the significant correlation in the self-portraits of changes in three elements of style with adverse events consistent with low mood.

Empirical analysis of contrast, colour, fractal dimension and productivity, quantitative and qualitative, in Rembrandt’s self-portraits and portraits do not support diagnoses of unipolar or of bipolar depression as clinically defined nor of neurodegenerative disease or cognitive deterioration due to aging. The results suggest that he experienced periods of low mood which can be characterised as normal grieving and response to adversity. The periods were short, did not affect him in any functional domain and coincided with increased productivity. The long duration of the mid-career change in contrast, the absence of significant change in use of colour aside from yellow, the short duration of comparatively small increases in fractal dimension, the long-term increase in productivity, quantitative and qualitative, and the absence of other symptoms of MDD or BPD including anhedonia, cognitive impairment, psychomotor disturbance, loss of energy, low self-esteem and functional impairment, all weigh against such diagnoses. The evidence is that Rembrandt remained healthy, active, creative, and productive throughout his life (3, 12, 200).

There is evidence of age-related macular degeneration but limited in its effects to a shift on the tritan spectrum. He appears to have aged normally with vision otherwise unimpaired. There is also evidence of a gradient in salience and complexity higher in self-portraits than in portraits of related and unrelated others consistent with experimental evidence. This is also consistent with self-portraits revealing more of the artist’s psychological state than other subjects. While the late self-portraits depict him as old they are not negative images. In one he paints himself as Xeuxis, the greatest painter of antiquity, said to have died laughing (1663). In others he depicts himself as St Paul (1663) and at an easel with Giotto’s perfect circles in the background (1663-69). These suggest a well-balanced man who has found spiritual peace and fulfilment of his ambition of artistic mastery.

### Limitations

This study is based on digital images of paintings not the paintings themselves. In addition, the images were not generated on a consistent basis. While all of the images are of high quality, they were produced under various lighting conditions and using varying equipment although there is reason to believe that this variation in fact enhances overall reliability of results by reducing the likelihood of any one format biasing the data (189). Only three elements of style were examined, and colour analysis is limited by the inability of devices to perceive colour as the eye does. The paintings examined do not include history, landscape or genre paintings nor any works on paper. While the sample, as a proportion of Rembrandt’s painted oeuvre is large (≌ 50%) the sample size of the self-portraits and portraits are each small from a statistical perspective. This constraint together with asymmetric distribution compelled the use of statistical techniques which have inherently less power than those used in regard to regular distributions. Stylistic examination of Rembrandt’s entire output might yield different results and would be a useful extension of research. Nevertheless, the results are consistent with biographical and aesthetic analysis.

## Data Availability

The raw data supporting the conclusions of this article will be made available by the authors, without undue reservation.

## Footnotes

1 For images of Rembrandt’s self-portraits in chronological order see: https://www.wga.hu/html_m/r/rembrand/27self/index.html

2 The years in which these events occurred are referred to here as Critical Years and highlighted in the figures below.

3 These include: temporal veins, periorbital structures, brow, inter-pupillary distance, wrinkles, the droop of his eyelids, nasolabial folds, marionette lines, submalar hollows, jowl formation and accumulation of neck fat.

4 For a detailed explanation of colour extraction using MatLab (see: 196).

5 See e.g.: https://www.reuters.com/article/us-netherlands-rembrandt-idUSKCN1IH14V.
